# Everolimus for Compassionate Use in Multiple Basal Cell Carcinomas

**DOI:** 10.1155/2013/604301

**Published:** 2013-09-23

**Authors:** Laura Eibenschutz, Delia Colombo, Caterina Catricalà

**Affiliations:** ^1^Dipartimento Clinico Sperimentale di Dermatologia Oncologica, Istituto Santa Maria e San Gallicano IRCCS, Via Fermo Ognibene 23, 00144 Roma, Italy; ^2^Novartis Farma Italia, 20065 Origgio, Varese, Italy

## Abstract

Everolimus is an inhibitor of the mammalian target of rapamycin (mTOR) and has been shown to have antineoplastic activity in addition to its use as an immunosuppressive agent for the prevention of organ transplant rejection. We report the use of everolimus for the compassionate treatment of four elderly, nontransplant patients presenting with multiple basal cell carcinomas (BCC). All patients had a long history of BCC, had refused surgery as a current treatment option, and did not respond to alternative treatments (including topical 5-fluorouracil and imiquimod). Patients were treated with oral everolimus (1.5–3.0 mg daily) for 12 months or longer: a complete and sustained response was seen in one case, and partial responses were seen in two other cases. Everolimus was well tolerated in these elderly patients. These promising preliminary data suggest that further dose-finding, controlled clinical studies are warranted to evaluate the antineoplastic effects of everolimus in patients affected by BCC who cannot or will not undergo surgery.

## 1. Introduction

The protein kinase family is involved in many critical regulatory cell functions. One pathway that plays a key role in signal transduction involves phosphatidylinositol-3-kinase (PI3K), which activates a cascade of other kinases, eventually providing a signal for cell proliferation. The downstream effector of PI3K is the mammalian target of rapamycin (mTOR), which plays a key regulatory role in protein translation by modulating the activity of other kinases via phosphorylation [1, 2]. Two agents that target mTOR are now commercially available: sirolimus and its derivative everolimus, and both drugs are approved for use as part of immunosuppressive regimens following renal or heart transplantation. In addition to their immunosuppressive properties, these proliferation signal inhibitors (PSIs) also have substantial antineoplastic activity, resulting from a number of different mechanisms. Firstly, there is now evidence that growth factors and oncogenic proteins can activate PI3K, and this leads, via mTOR, to the phosphorylation and activation of a number of protein kinases that promote cancer cell proliferation and survival. Inhibition of mTOR activity can block this signaling cascade and thus interfere with tumor cell proliferation [3]. Secondly, vascular endothelial cell proliferation and angiogenesis are under the control of vascular endothelial growth factors (VEGFs), which act, at least in part, through the PI3K-mTOR signaling pathway. By blocking this pathway, PSIs may also exert antineoplastic activity by interfering with tumor angiogenesis [4]. Thirdly, by inhibiting VEGF-C, PSIs may also inhibit lymphangiogenesis and lymphatic metastases as has been shown in experimental models [5]. Finally, PSIs may modulate the levels of hypoxia-inducible factors, key regulators of the cancer cell response to hypoxia [6].

The anticancer effects of PSIs have been preliminarily investigated in renal transplant recipients, with some evidence of efficacy in inducing regression or stabilization of lymphoma [7], Kaposi's sarcoma [8], and skin cancer [9]. We report here four cases of the compassionate use of everolimus in nontransplant recipients with recurrent multiple basal cell carcinomas (BCC) that were resistant to conventional therapy. Patients gave their informed consent, and the study protocol was approved by the local Ethic Committee.

## 2. Case Reports

*Patient 1*. A male patient who was 94 years old presented at our institution with multiple facial BCC, comprising a nodular BCC on the nose and two other superficial lesions. The patient refused surgical ablation of the lesions and was thus submitted to pharmacological treatment with topical immunomodulators (including topical 5-fluorouracil and imiquimod). Complete response was achieved for all lesions following 12 weeks of therapy, but after 1 year the nasal lesion relapsed and a suspect keratoacanthoma appeared on the forehead. A further 8-week course of imiquimod was administered, but the nasal BCC increased in size. Everolimus (1.5 mg daily *per os*) was initiated in August 2006, increased to 3 mg daily after 3 months, and continued for a total treatment period of 12 months. Following an initial ulcerative phase, after 8 months of everolimus treatment, the nasal BCC disappeared completely. The forehead lesion remained unchanged, and it was surgically removed and subsequently shown to be an invasive squamous cell carcinoma. The complete response of the nasal BCC persisted for the last follow-up, 8 months after discontinuation of everolimus. Subsequently the patient died for natural cause.

*Patient 2*. A female patient aged 85, who had in the last few years undergone several interventions to surgically remove multiple BCC, presented in January 2004 with 3 facial BCC: one nodular lesion on the nose and two superficial lesions on the forehead. The patient refused further surgeries and was therefore treated with topical 5-fluorouracil, with complete and sustained response of the superficial lesions, but no response of the nodular lesion on the nose. From September 2006, everolimus was administered at an initial dose of 1.5 mg daily, which was raised to 3 mg daily after two months and continued for a total of 18 months. No response of the nasal lesion was observed: the size was unchanged after 3 months of treatment and slightly increased after 6 months. No new lesions appeared. In December 2007 a suspected keratoacanthoma appeared under the right eyelid and required surgical removal. This was diagnosed as a microinvasive squamous cell carcinoma. During this surgical intervention, the nasal lesion was also removed and the diagnosis of BCC was confirmed. Wound healing required more than 2 months and in April 2008 a rapidly growing new BCC lesion on the mandibular region appeared, with surgical indication. Subsequently the patient died for natural cause. 

**Figure 1:**
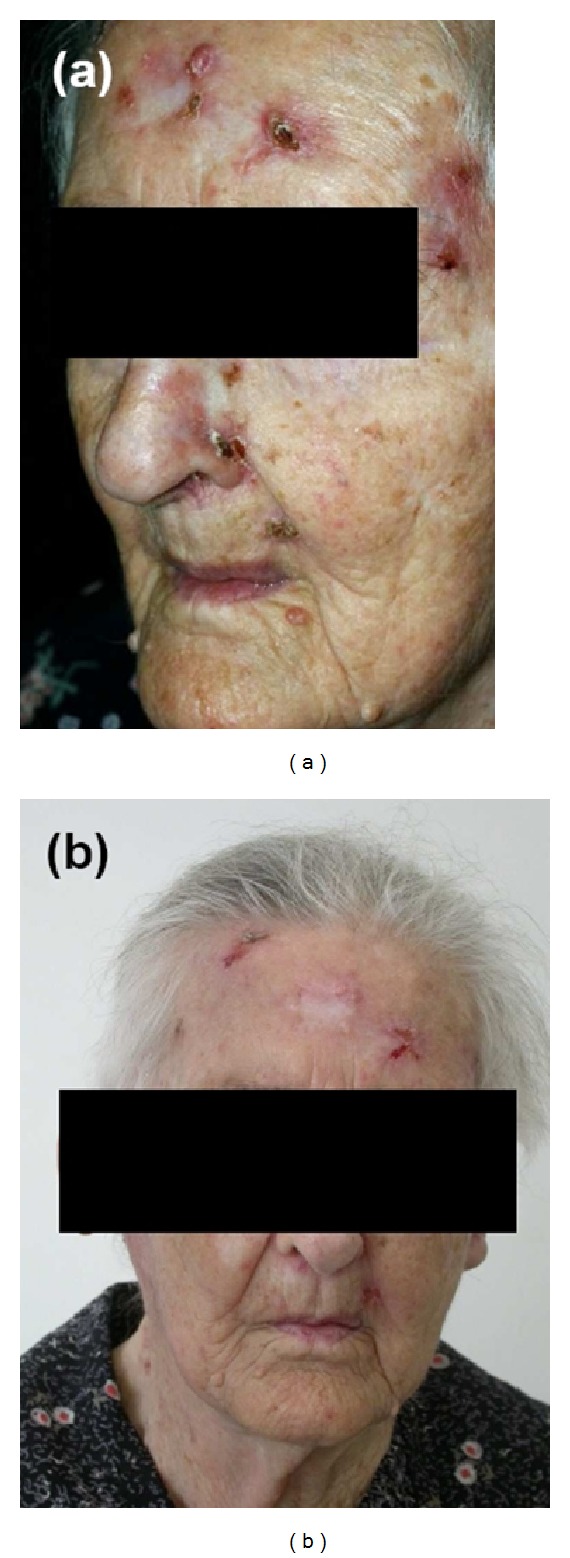
Patient 3. Forehead basal cell carcinoma lesions before everolimus treatment (a) and after six months of everolimus 3 mg daily (b).

*Patient 3*. This female patient, aged 91 years, had undergone several interventions to remove multiple BCC over the previous 10 years. In the last 5 years, the patient developed partially ulcerated superficial BCC lesions of both left and right external and internal acoustic meatuses and several superficial BCC on the forehead and in the nuchal region. The patient consistently refused surgery, and over a period of a few years she had lost a large part of the right auricle. Topical application of 5-fluorouracil was prescribed for the meatal lesions and cryotherapy with liquid nitrogen for the other lesions. The skin BCC underwent complete remission, whereas the meatal BCC only partially responded to topical therapy. The clinical picture remained stable for some years; then at a follow-up visit, an extensive progression of the acoustic meatus lesion of the right ear was observed in addition to the occurrence of new facial lesions with a mean diameter of 3 cm. The patient again refused surgery, so everolimus was started at 2.5 mg daily (rapidly increased to 3 mg daily) from September 2006 until September 2008. Once everolimus administration was started, the forehead lesions improved, while all other lesions remained unchanged (Figure 1). At the 24 months follow-up visit, the right ear BCC was in progression, while the forehead BCC were stable. Subsequently the patient died for natural cause. 

**Figure 2:**
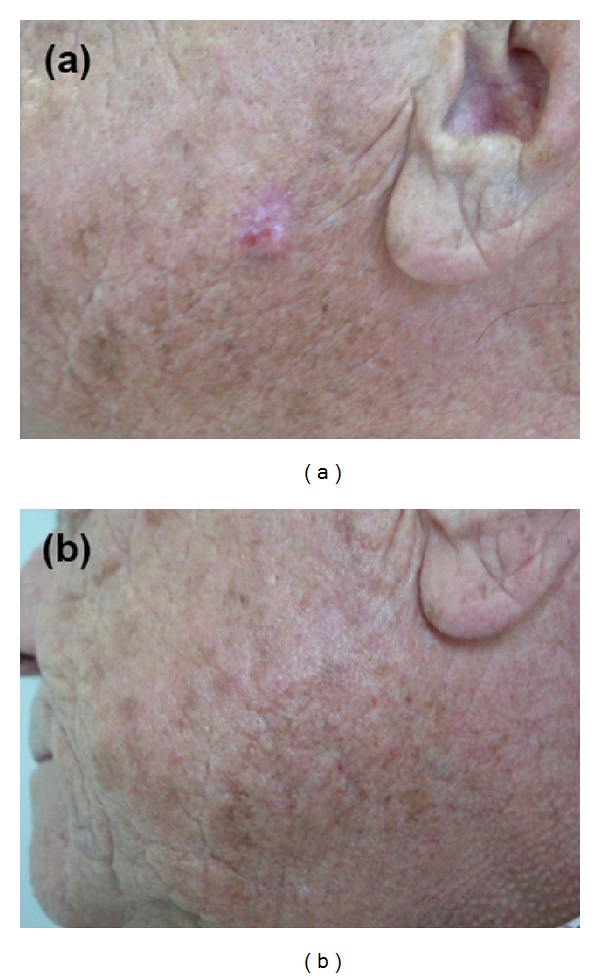
Patient 4. (a) A facial basal cell carcinoma appeared after six months of everolimus treatment. (b) The lesion had disappeared after a further nine months of treatment (3 mg daily).

*Patient 4*. This male patient (aged 73 years), who had a 30-year history of multiple BCC with repeated surgical interventions, had developed more than 20 superficial and nodular BCC of the trunk and the face, some of them ulcerated, in the last two years. Having refused further surgical treatments, the patient was submitted to photodynamic therapy and topical application of imiquimod on the smallest lesions. Some of the superficial BCC lesions regressed under these treatments, while the nodular forms responded only partially, and new nodular lesions were observed at follow-up. At this point, the patient accepted surgery, but only for nodular BCC. Everolimus (3 mg daily) was started in June 2007 for the residual superficial lesions and is still ongoing after more than one year. The lesions remained stable until the follow-up visit in December 2007, when two new BCC (one on the face and one on the ear) were observed. At the follow-up visit, in September 2008, the newest lesions had disappeared (Figure 2). At the last follow-up visit, in November 2012, the clinical profile was stable. 

*Blood Chemistry*. No clinically relevant changes in hematology or blood chemistry parameters were observed in all patients, except for a slight and transient increase in total cholesterol (Table 1).

## 3. Discussion

The PSI everolimus can induce the remission of nonmelanoma skin cancer in kidney and heart transplant recipients [9, 10], while its effect on nonmelanoma skin cancer in nontransplant patients has not been previously investigated. In our four elderly patients with a long history of relapsing multiple BCC, who refused surgical treatment and were resistant to previous topical treatments, everolimus was proposed for compassionate use. Everolimus was administered cautiously at low dose, bearing in mind the advanced age of the patients and the off-label indication. Tolerability was generally good: no adverse events were reported, with the exception of patient 1, where nausea and dyspepsia occurred initially, when everolimus was administered after a period of fasting. These adverse effects diminished significantly when the drug was administered after meals. The response of BCC lesions to everolimus was variable: a complete and sustained response was seen in patient 1, and partial responses were observed in patients 3 and 4, while in patient 2 everolimus had no effect. Patients 2 and 3 had a long history of occupational exposure to ultraviolet radiation, but there were no other significant precipitating factors such as immunosuppression or arsenic exposure. The positive results were obtained in patients with multiple BCC, while no response was observed in squamous cell carcinomas. The preliminary evidence of efficacy of everolimus seen in these cases, where low doses were employed in patients of advanced age with a long history of disease not responding to alternative treatments, indicates that everolimus may warrant further investigations for BCC in nontransplant patients. The doses used in these four patients (1.5–3.0 mg daily) were considerably lower than the 10 mg/day dose approved by the FDA in March 2009 for the treatment of patients with advanced renal cell carcinoma [11] and used in phase II clinical trials in other cancer indications (5–10 mg/day) including breast [12], pancreatic [13], and neuroendocrine tumors [14]; consequently, it is possible that higher doses may be more effective for BCC also. One important, although preliminary, observation was the good tolerability of everolimus when administered to these elderly patients at up to 3 mg/day, with only a few, slight, and transient adverse events noted. Further dose-finding and controlled clinical studies need to be conducted to evaluate the possible antineoplastic effect of everolimus in skin cancers in patients affected by BCC who cannot or will not undergo surgery.

## Figures and Tables

**Table 1:** Blood chemistry and haematology parameters assessed at baseline and end of treatment with everolimus.

	Patient 1		Patient 2		Patient 3		Patient 4	
	Baseline	End of treatment	Baseline	End of treatment	Baseline	End of treatment	Baseline	End of treatment
Creatinine (mg dL^−1^)	1.00	1.20	0.90	0.80	0.84	0.81	1.13	0.90
Triglycerides (mg dL^−1^)	153	198	50	91	82	118	100	117
Cholesterol (mg dL^−1^)	195	195	167	207	138	175	195	203
Haemoglobin (g dL^−1^)	13.0	10.0	12.0	12.5	10.0	9.2	14.5	13.6
Haematocrit (%)	38.0	30.0	37.0	37.1	32.0	27.0	41.7	41.7
Platelets (×10^3^ *µ*L^−1^)	261	238	280	360	151	238	269	245
